# Role of trehalose in heat and desiccation tolerance in the soil bacterium *Rhizobium etli*

**DOI:** 10.1186/1471-2180-12-207

**Published:** 2012-09-17

**Authors:** Mercedes Reina-Bueno, Montserrat Argandoña, Joaquín J Nieto, Alba Hidalgo-García, Fernando Iglesias-Guerra, María J Delgado, Carmen Vargas

**Affiliations:** 1 Department of Microbiology and Parasitology, Faculty of Pharmacy, University of Seville, Profesor García González 2, Seville, 41012, Spain; 2 Department of Organic and Pharmaceutical Chemistry, Faculty of Pharmacy, University of Seville, Profesor García González, Seville, 41012, Spain; 3 Estación Experimental del Zaidín, CSIC, PO Box 419, Granada, 18080, Spain

## Abstract

**Background:**

The compatible solute trehalose is involved in the osmostress response of *Rhizobium etli*, the microsymbiont of *Phaseolus vulgaris*. In this work, we reconstructed trehalose metabolism in *R. etli*, and investigated its role in cellular adaptation and survival to heat and desiccation stress under free living conditions.

**Results:**

Besides trehalose as major compatible solute, *R. etli* CE3 also accumulated glutamate and, if present in the medium, mannitol. Putative genes for trehalose synthesis (*otsAB/treS/treZY*), uptake (*aglEFGK*/*thuEFGK*) and degradation (*thuAB*/*treC*) were scattered among the chromosome and plasmids p42a, p42c, p42e, and p42f, and in some instances found redundant. Two copies of the *otsA* gene, encoding trehalose-6-P-synthase, were located in the chromosome (*otsAch*) and plasmid p42a (*otsAa*), and the latter seemed to be acquired by horizontal transfer. High temperature alone did not influence growth of *R. etli,* but a combination of high temperature and osmotic stress was more deleterious for growth than osmotic stress alone. Although high temperature induced some trehalose synthesis by *R. etli*, trehalose biosynthesis was mainly triggered by osmotic stress. However, an *otsAch* mutant, unable to synthesize trehalose in minimal medium, showed impaired growth at high temperature, suggesting that trehalose plays a role in thermoprotection of *R. etli*. Desiccation tolerance by *R. etli* wild type cells was dependent of high trehalose production by osmotic pre-conditioned cells. Cells of the mutant strain *otsAch* showed ca. 3-fold lower survival levels than the wild type strain after drying, and a null viability after 4 days storage.

**Conclusions:**

Our findings suggest a beneficial effect of osmotic stress in *R. etli* tolerance to desiccation, and an important role of trehalose on the response of *R. etli* to high temperature and desiccation stress.

## Background

Rhizospheric rhizobia are subjected to fluctuating osmotic, heat and drought stresses due to the succession of drought and rain periods, the exclusion of salts like NaCl from root tissues, the release of plant exudates, or the production of exopolymers by plant roots and other rhizobacteria. In addition, rhizobia must also adapt to osmotic and oxidative stresses during the infection process and in a nodule exchanging nutrients with the host plant. On the other hand, drought stress is particularly important when rhizobia are used as inoculants for legumes, since it affects cell viability during storage in carrier-based (i.e. seed-coated) inoculants [1,2]. Therefore, besides symbiotic efficiency, improvement of survival of rhizobia under conditions of the above abiotic constraints may constitute a competitive trait for either native or inoculant rhizobia, to persist in soil and solid inoculant formulations, and to improve the colonization and/or infection process. The responses to osmotic, drought and heat stress in bacteria involve very complex adaptation mechanisms, but one common element of the three responses is the synthesis of protectant molecules named compatible solutes [3]. Indeed, the role of compatible solutes goes beyond osmotic adjustment alone to protection of cells and cell components from freezing, desiccation, high temperature, and oxygen radicals, as well as to serve as sources of carbon, energy and nitrogen [4].

Trehalose (*O*-α,-D-glucosyl-[1→1]-α-D-glucoside) has been found as the main compatible solute in almost any rhizobial strain tested so far, and its accumulation has been detected in free-living cells, bacteroids, and nodules [2,5-8]. Trehalose accumulation by *R. leguminosarum* bv *trifolii* and *Sinorhizobium meliloti* reaches its maximal levels at stationary phase of growth [5,7,9]. Out of the five different routes known for trehalose biosynthesis, three pathways have been found in rhizobia. First, the OtsA-OtsB route, which is very well conserved among insects, plants, fungi and bacteria, involves the transfer of glucose from UDP-glucose to glucose-phosphate to form trehalose-6-phosphate by trehalose-6-phosphate synthase (OtsA). Then, a trehalose-6-phosphate phosphatase (OtsB) dephosphorylates this intermediate to produce trehalose [2,5,7,10]. Second, trehalose synthase (TreS), first described in mycobacteria [11], catalyzes the reversible conversion of maltose and trehalose. In the case of *Bradyrhizobium japonicum*, trehalose is accumulated to a greater extent in a *treS* mutant, suggesting that TreS is involved in trehalose degradation to maltose [2]. A third pathway first discovered in *Rhizobium* sp. M-11 [12] and the archaeon *Sulfolobus acidocaldarius*[13], converts the terminal unit of a linear maltodextrin (e.g., glycogen or starch) to trehalose via maltooligosyl trehalose synthase, encoded by *treY*, and maltooligosyl trehalose trehalohydrolase (TreZ).

Apart from stress protectant, trehalose also serves as a carbon and energy source for many bacteria, including rhizobia. In soil, trehalose originates from nodules during nodule senescence [14] and as an excretion product from fungi [15]. There are several known pathways for trehalose catabolism in microorganisms. The major enzyme involved in the turnover of trehalose, trehalase (α,α,1,1-glucosyl hydrolase), usually belongs to families 37 and 15 of glycoside hydrolases [16,17]. In other cases, trehalose degradation involves a trehalose phosphorylase (TreP) [18]. Degradation of trehalose-6-phosphate can be mediated by a trehalose 6-phosphate hydrolase (TreC), belonging to family 13 of glycoside hydrolases [16], or a trehalose-6-phosphate phosphorylase (TrePP) [19].Trehalase, trehalose phosphorylase, and trehalose-6-phosphate hydrolase were detected in soybean nodules formed by *B. japonicum*[20], but orthologous genes for these enzymes were not found in the genome of *S. meliloti*[21]. In the former species, two ABC transport systems (ThuEFGK and AglEFGAK), and one major catabolic pathway (ThuAB) have been reported for trehalose [22,23].

In rhizobia, the effect of trehalose accumulation on tolerance to osmotic and drought stress, as well as symbiotic performance, appears to be dependent on the particular stress, the rhizobial species, and the host genotype. Regarding osmotic stress, OtsAB seems to play a major role in trehalose accumulation under hyperosmotic conditions, and it is the main system involved in osmoadaptation of *S. meliloti*[5] and *B. japonicum*[2]. In addition, accumulated trehalose seems to have a major role in protecting *B. japonicum*[24] and *R. leguminosarum* bv *trifolii*[7] against desiccation stress. With respect to symbiotic phenotype, in *B. japonicum* trehalose accumulation is involved in the development of symbiotic nitrogen-fixing root nodules on soybean plants [2]. In contrast, in other rhizobia such as *R. leguminosarum* bv *trifolii* or *S. meliloti*, trehalose accumulation has been proposed to be important only for competitiveness [5,7]. The role of trehalose as thermoprotectant has been established in yeast [25] and bacteria such as *E. coli*[26], *Salmonella enterica* serovar Typhimurium [27] or the halophilic bacterium *Chromohalobacter salexigens*[28]. However the role of trehalose in protection against heat stress in rhizobia has not yet been investigated.

Common bean (*Phaseolus vulgaris*) is an important crop in the diet of people of Latin America. In this region, it is mainly nodulated by *R. etli*[29]. The complete genome sequence of *R. etli* CFN 42 has been reported ( http://www.ccg.unam.mx/retlidb/) [30]. It contains more replicons (a circular chromosome and six large plasmids) than any other completely sequenced nitrogen-fixing bacterium, but several pieces of evidence suggest an exogenous origin for plasmids p42a and p42d

Suarez and co-workers [10] reported an *otsA* mutant still capable of accumulating trehalose to a certain extent, which was nevertheless osmosensitive and displayed reduced nodulation and lower nitrogenase activity, and consequently reduced plan biomass. In contrast, an *OtsA* overexpressing *R. etli* strain showed increased trehalose content and was more tolerant to osmotic stress than the wild-type. Bean plants inoculated with the OtsA overexpressing strain showed improved nodulation and nitrogen fixation, and increased drought tolerance. In addition, transcriptomic analysis of plant nodules in symbiosis with the OtsA overexpressing strain revealed induction of several genes involved in stress tolerance, and carbon and nitrogen metabolism, suggesting that trehalose (or a trehalose derivative) plays a role as a signal molecule from rhizobia to the legume nodule [10].

Whereas increased trehalose levels in *R. etli* inoculant strains seem to favor drought tolerance of the host legume, the involvement of trehalose in desiccation tolerance of *R. etli* free-living cells has not been investigated. In this work, we address the role of trehalose in heat and desiccation tolerance of this soil bacterium. Based on genome analysis, we reconstructed the *R. etli* trehalose metabolism, and found evidence for a horizontal transfer origin of the *otsA* copy located in plasmid p42a. In addition, we showed that inactivation of the chromosomal copy of *otsA* (*otsAch*) completely abolishes trehalose synthesis by *R. etli* in mannitol minimal medium. Finally, we showed an important role for trehalose in thermoprotection and desiccation tolerance of *R. etli* free-living cells.

## Methods

### Bacterial strains, plasmids and culture conditions

The bacterial strains and plasmids used are listed in Table 1. *R. etli* CE3 (a spontaneous Sm^r^ mutant of *R. etli* CFN 42^T^) [31] was used as the wild type strain. *R etli* strains were routinely grown in complex TY medium [32]. *E. coli* strains were grown aerobically in complex LB medium [33]. B^-^medium [34], which contains 10 g l^-1^mannitol as the sole carbon source, was used as minimal medium for *R. etli*. When appropriate, trehalose and glucose were also used as carbon source at a final concentration of 20 mM. Osmotic strength of this medium was increased by the addition of 0.1 to 0.2 M final concentration of NaCl. pH was adjusted to 7.2 (for TY) or 5 (for B^-^). Solid media contained 2% of Bacto agar (Difco). *E. coli* cultures were incubated at 37°C. *R. etli* cultures were incubated at 28°C or 35°C [29]. When used, filter sterilized antibiotics were added at the following final concentrations (μg ml^-1^): ampicillin (ap), 150 for *E. coli*; chloramphenicol, 30 for *E. coli*; gentamicin (Gm), 20 for *E. coli*, 25 for *R. etli*; streptomycin (Sm) 20 for *E. coli*, 40 for *R. etli*; spectinomycin (Spc) 80–100 for *R. etli* and nalidixic acid 20 for *R. etli*. When appropriate, the following compounds were added to the media (final concentration): X-Gal (5-bromo-4-chloro-3-indolyl-β-D-galactopyranoside, Sigma, 40 μg/ml), IPTG (isopropyl-β-D-1-thiogalactopyranoside, Sigma, 25 μg/ml). Growth was monitored as the optical density of the culture at 600 nm (OD_600_) with a Perkin-Elmer Lambda 25 UV/Vis spectrophotometer. 

**Table 1:** Bacterial strains and plasmids used in this study

**Strain or plasmid**	**Relevant genotype and/or description**	**Source or reference**
***R. etli*strains**		
CFN 42	Wild type	[29]
CE3	Spontaneous Sm^r^ mutant of *R, etli* CFN 42 Nal^r^	[31]
CMS310	*R. etli* CE3 *otsAch*::ΩNal^r^Sm^r^Spc^r^	This study
***E. coli*strain**		
DH5α	*supE44*Δ (*lac*)*U169* ϕ80d *lacZΔM15 hsdR17 recA1 endA1 gyrA96 thi-1 relA1*; host for DNA manipulations	[35]
**Plasmids**		
pSK(-)	Cloning vector; Ap^r^	Stratagene
pUC19-301	Cloning vector, Ap^r^	J. Onofre, Personal Communication
pHP45Ω	pBR322 derivative carrying the Ω cassette; Ap^r^Sm^r^Sp^r^	[36]
pRK600	Helper plasmid; Cm^r^*tra*	[37]
pJQ200-SK	Suicide vector; Gm^r^*mobsac*	[38]
pMotsA1	4.2-kb blunt fragment from *R. etli* CE3 genome (containing *frk*, *otsAch*, *pgi*) cloned into pUC19301 in *Eco*RV; Apr	This study
pMotsA4	4,1-kb *Bgl*II-*Xba*I fragment from pMotsA1 cloned into pSK in *Bam*HI-*Xba*I; Ap^r^	This study
pMotsA5	pMotsA4 derivative containing an *Bgl*II recognition site within *otsAch*; Ap^r^	This study
pMotsA6	pMotsA5 derivative with Ω casete within *otsAch*; Ap^r^Sm^r^Spc^r^	This study
pMotsA7	6.1-kb *Apa*I-*Xba*I fragment from pMotsA6 (containing *frk,otsAch*, *pgi*) cloned into pJQ200-SK; Gm^r^Sm^r^Spc^r^	This study

### Tolerance to desiccation

Aliquot volumes (1 ml) of B^-^ medium cultures in early stationary phase were harvested by centrifugation. Cell pellets were washed with the same medium without any carbon source, centrifuged for 5 min at 13000 rpm and, after removing the supernatant, vacuum dried. Two variations of the protocol described by Manzanera et al. [39] were used. In a first step, two replicates of all samples were dried by vacuum in a Memmert V0200 vacuum oven at 20°C and 313 mbar for 20 h. After that, for each sample, one replica was taken out from the oven, sealed and stored at 28°C, and the other was subjected to a further step under vacuum consisting on a temperature ramping of 2°C/min with a 15-min pause after every increase of 2°C, up to a maximum temperature of 30°C, followed by storage at 28°C. For assessment of viability, after variable time periods, dried samples were resuspended in 1 ml of TY complex medium, and serial dilutions were plated on TY plates, incubated at 28°C, and counted to determine CFU. Viability was measured before (taken as 100% survival) and just after drying, and at 4 days, 1, 2, and 3 weeks storage, and expressed as percentage of viable cells.

### Extraction and determination of intracellular solutes by ^13^C-NMR spectroscopy

*R. etli* wild-type and *otsA* mutant strain (CMS310) were grown in B^-^medium with 0.2 M NaCl at 28°C until early-stationary phase. Cells were collected by centrifugation and washed with the same medium without any carbon source. Cell pellet was resuspended in 10 ml of extraction mixture (methanol:chloroform:water; 10:5:4) and extracted by gently shaking for 30 min at 37°C. Cell debris was removed by centrifugation, and supernatants were extracted once with chloroform:water (1:1) and freeze-dried. The solids were dissolved in D_2_O (0.6 ml). ^13^C-NMR spectra were recorded at 25°C on a Brucker AV500 spectrometer at 125 MHz. The chemical shifts are reported in ppm on the *δ* scale relative to tetramethylsilane. Signals were assigned by comparison with previously published chemical shift values [6] and compared with ^13^C-NMR of pure compounds.

### Extraction and determination of intracellular trehalose content

Trehalose determination was performed basically as described by Blazquez et al. [40] by the following procedure. For free-living cells, pellets from 15 ml of early stationary phase cultures in B^-^medium were washed with isotonic carbon-free medium and resuspended in 1 ml of the same medium. Cells were lysed by 30 min of incubation at 95°C and, after centrifugation, the supernatant was used to determine the trehalose content in a total volume reaction of 200 μl containing 100 μl of the supernatant, 90 μl of 25 mM sodium acetate buffer (pH 5.6) and 0.02 U of trehalase (Sigma). For each sample, endogenous glucose was monitored by performing a parallel reaction in which trehalase was substituted by water. After overnight incubation at 37°C, the glucose released by trehalose hydrolysis was determined by adding 150 μl of the previous reaction to 150 μl of a mixture of 0.66 mg ml^-1^*Aspergillus niger* glucose oxidase (Sigma), 0.25 mg ml^-1^ horseradish peroxidase in 0.5 M phosphate buffer, pH 6.0 (Sigma), and 50 μl of 2.33 mg ml^-1^ o-toluidine (Panreac). After 30 min of incubation at 37°C, 1.5 ml of water was added to the samples and absorption was measured at 420 nm in a Perkin Elmer Lambda 25 UV/Vis spectrophotometer. Values were compared to those obtained from stock solutions of glucose standards in a concentration range of 0 to 1000 μgml^-1^. Finally, trehalose content was calculated from the glucose content by performing a standard curve with commercial trehalose (Sigma) ranging from 1 to 5 mM. Trehalose concentration was expressed as μmol mg protein^-1^. Nodules were fractionated into bacteroids and nodule cytosol as described by Delgado et al. [41]. Trehalose content was determined colorimetrically as described above.

### Determination of protein content

The same cultures were used for determination of both trehalose and protein content. 1 ml aliquots were taken at early stationary phase and cell protein content was determined in triplicate by using a bicinchoninic acid (BCA) proteinassay kit (Pierce) as described by García-Estepa et al. [42].

### Methods for nucleic acid manipulation and construction of a *R. etli otsA* mutant

Plasmid DNA was isolated from *E. coli* with a Wizard Plus SV miniprep kit (Promega), and genomic DNA was isolated with a SpinClean Genomic DNA Purification kit (Mbiotech). Restriction enzyme digestion and ligation were performed as recommended by the manufacturers (Amersham-Pharmacia Biotech and Fermentas). DNA sequencing was performed by Newbiotechnic (Seville, Spain). To generate the *R. etli* CE3 *otsAch* mutant CMS310 (*otsAch*::Ω), a 4.119-bp fragment from the *R. etli* genome containing 394-bp of the adjacent gene *frk*, *otsAch* and 1.488-bp of the *pgi* gene, was amplified with *Pfu* Turbo DNA polymerase (Stratagene) by using two synthetic oligonucleotides (*otsA*^R^-FW: 5’-AAGACGGCTGTGAACGACGAG-3’ and *otsA*^R^-RV: 5’-CAAATCCGACATCGTCAAATTCTC-3’). The resulting PCR fragment was cloned into pUC19-301 digested with *Eco*RV to obtain the plasmid pMOtsA1. This plasmid was digested with *Bgl*II-*Xba*I, to remove the 4.2-kb fragment containing the *otsA* region which was cloned in pSKbluescript previously digested with *Bam*HI-*Xba*I to obtain the plasmid pMotsA4. Subsequently, a *Bgl*II recognition site was generated in *otsAch* gene sequence, using the PCR-based QuickChange Site Directed Mutagenesis Kit (Stratagene) and the primers: *otsA*^R^*Bgl*II FW (5’-GAAGAGAGGGCATTGGCGA**AGATCT**CGGCAACGGATTGTTCGATTC-3’), and otsA^R^*Bgl*II RV (5’-GAATCGAACAATCCGTTGCCG**AGATCT**TCGCCAATGCCCTCTCTTC-3’), that were modified (residues underlined) to generate the corresponding restriction site (in bold), to obtain the plasmid pMotsA5. To interrupt the *otsA* gene, the resulting plasmid was linearized with the enzyme *Bgl*II and ligated to a 2-kb *Bam*HI fragment obtained from pHP45-Ω plasmid [38], containing the Ω interposon for insertional mutagenesis (Sm^r^). The resulting plasmid was designated pMotsA6. To recombine the *otsA* mutation into the *R. etli* chromosome, a 6.1-kb *Apa*I-*Xba*I fragment from pMotsA6 was cloned into the suicide vector pJQ200-SK (Gm^r^) [38] to obtain plasmid pMotsA7, which was mobilized into the *R. etli* CE3 by triparental mating. Mutant strains resulting from a double homologous recombination event were identified as Spc^r^Gm^s^ colonies on TY plates containing 10% sucrose. One of these colonies was purified for further analysis and was designated CMS310 (*otsAch*). Insertion of the omega cassette in CMS310 was confirmed by PCR and sequencing.

### Conjugal transfer of plasmids

Plasmids were transferred from *E. coli* to *R. etli* by triparental mating on TY medium, using pRK600 as a helper plasmid [37], as described by Vargas et al. [43] but with a 1:2:1:(donor:receptor:helper) ratio.

### Sequence and phylogenetic analyses

The sequence of the *R. etli* CFN 42 genome is available at NCBI microbial genome database ( http://www.ncbi.nlm.nih.gov/genomes/lproks.cgi; Ac N°: NC_007761), and at http://www.ccg.unam.mx/retlidb/. Sequence data were analyzed using BLAST (NCBI http://ncbi.nlm.nih.gov/BLAST). ORF assignments of the metabolic pathways more relevant for this work was performed by comparing the information available at the Kyoto Encyclopedia of Genes and Genomes (KEGG) [44] and MetaCyc [45]. Codon preference was analysed at the Kasuza Codon Use Database ( http://www.kazusa.or.jp/codon/). Phylogenetic and molecular evolutionary analyses were conducted using MEGA version 5 [46]. Sequences were aligned with ClustalW (1.6) using a BLOSUM62 matrix, and manually edited. The phylogenetic tree was inferred using the Neighbor-Joining method [47], and the evolutionary distances were computed using the Poisson correction method. The rate variation among sites was modelled with a gamma distribution (shape parameter = 1) and all positions containing gaps and missing data were eliminated only in pairwise sequence comparisons. The robustness of the tree branches was assessed by performing bootstrap analysis of the Neighbour-Joining data based on 1000 resamplings [48].

### Plant inoculation, growth conditions, and drought treatment

Seeds of *Phaseolus vulgaris*, var. Negro jamapa were surface sterilized in 96% (v/v) ethanol for 30 s, followed by 5% sodium hypochlorite for 5 min and then thoroughly washed five times with sterilized water [41]. Sterilized seeds were incubated two hours in sterile distilled water in darkness. The imbibed seeds were deposited on plates containing 1% water-agar, and let them to germinate at 30°C during 60 h. Three day after sowing, selected uniform seedlings were planted in sterile 2-kg pots (4/pot) containing a mixture of vermiculite/sand (1:1 v/v) as substrate. Each seedling was inoculated with 1 ml of the strains culture of *R. etli* wild-type and *otsA* mutant strain (CMS310) containing 10^8^ cells/ml at the log-phase of growth. Plants were grown in controlled environmental chambers (night/day temperature 19/25°C, photoperiod 16/8 h, PPF 400 μmol m^-2^ s^-1^ and relative humidity 60 to 70%) [49]. Plants were watered with N-free mineral solution [50] and water alternately. When plants were three weeks old, they were randomly separated into two sets: control and drought stress. Drought stress was imposed by withholding water for 5 days (moderate drought) or for 10 days (severe drought). Control plants were supplied daily with nutrients solution to field capacity. Leaf water potential (Ψ_w_) was measured in the first fully expanded leaf of common bean plants with C52 sample chambers connected to a HR-33 T psychrometer (Wescor. Inc., Logan UT, USA).

### Plant and nodule biomass, nitrogen content and nitrogen-fixation assays

Plant and nodule dry weight were determined after drying fresh plant material that was heated at 60°C for 48 h. Total nitrogen content per plant was determined by the Kjeldahl method [51]. Nitrogenase activity was analyzed by using the acetylene reduction activity (ARA) assay. A Hewlett-Packard model 5890 gas chromatograph (Agilent Technologies, S.L., Madrid) equipped with a flame ionization detector was operated with a molecular sieve 5A (60 to 80 mesh) column (180 × 0.32 cm). N_2_ at 60 ml min^–1^ served as a carrier gas. Oven, injector, and detector temperatures were 60, 90, and 110°C, respectively. Nodules (0.3 g) were placed in 17-ml tubes that were filled with 10% acetylene. Gas samples (0.5 ml) were taken from the tubes for ethylene analyses after incubation for 10 and 20 min. Concentration of ethylene in each sample was calculated from standards of pure ethylene.

### Leghemoglobin content

Leghemoglobin content was measured by fluorimetry as previously described by La Rue and Child [52]. Nodules (0.3 g) were ground with 4 ml Lb extraction buffer (Na_2_HPO_4_·2H_2_O 40 mM (pH 7.4); NaH_2_PO_4_·H_2_O 10 mM (pH 7.4); K_3_Fe (CN)_6_ 0.02%; NaHCO_3_ 0.1%) supplemented with 0.1 g polyvinylpolypirrolidone (PVPP). The homogenate was centrifuged at 12 000 rpm at 4°C for 20 min, to retain the supernatant. Clear supernatant (50 μl) and saturated oxalic acid (3 ml) were mixed in screwcapped tubes, which were sealed and autoclaved for 30 min at 120°C and then let to cool to room temperature. The fluorescence of the solutions was measured with a Shimadzu (Shimadzu Scientific Instruments, Kyoto, Japan) spectrophotofluorometer equipped with a mercury-xenon lamp and a RF-549 red-sensitive photomultiplier. The excitation wavelength was 405 nm and the emission monochromator setting was 650 nm. The difference in fluorescence between heated and unheated samples was proportional to haem protein concentration.

## Results

### Trehalose synthesis by *R.etli* is triggered mainly by salinity stress

**Figure 1:**
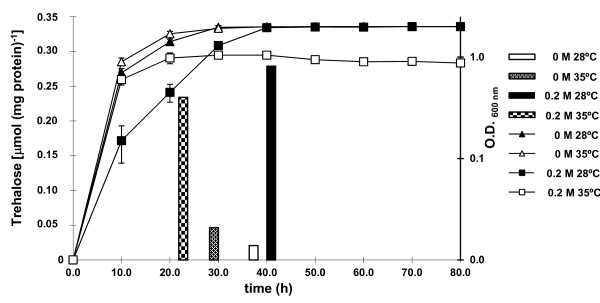
**Growth and accumulation of trehalose by *R. etli* in response to high temperature and salinity stress.** Cells were grown in mannitol minimal medium B^-^ at 28°C and 35°C, with 0.0 and 0.2 M NaCl, up to early stationary phase. Trehalose content was measured colorimetrically as described in Materials and Methods. For each determination, a growth curve under the same condition used to measure trehalose accumulation is shown. Histograms representing trehalose accumulation are shown above the sampling time. The trehalose values are the mean of three replicas of each condition in two independent experiments ± SD (standard deviation).

Heat stress induces accumulation of trehalose in yeasts [25] and bacteria such as *E. coli*[26] or *Salmonella typhi* serovar Typhimurium [27]. In rhizobia, including *R. etli*[10], trehalose synthesis has been shown to be stimulated by salinity, but its role against heat stress has not been yet tested. In this study, we compared the influence of salinity and high temperature on growth and trehalose accumulation in *R. etli*. For this purpose, *R. etli* wild-type strain was grown up to early stationary phase in B^-^minimal medium alone or with 0.2 M NaCl, at 28°C and 35°C, and trehalose content was determined colorimetrically as described in Materials and Methods. As shown in Figure 1, osmotic stress alone caused a delayed growth, but high temperature alone did not influence growth of *R. etli*. However, growth of cells subjected to both stresses was more impaired than that of cells grown under osmotic stress alone, showing an attenuated exponential phase, and reaching final O.D_600_ values below 0.9. As shown in Figure 1, under non stress conditions, trehalose levels in *R. etli* were below 0.025 μmol/mg protein. To determine trehalose content in response to high temperature stress, we compared the accumulation of trehalose at 28°C and 35°C in cells grown without NaCl added. Under these conditions, trehalose accumulation by *R. etli* cells increased by 2.2-fold, but trehalose levels remained very low. However, a pronounced response in trehalose accumulation was observed due to salinity stress at both temperatures. Thus, trehalose levels in cells grown in minimal medium with 0.2 M NaCl at 28°C and 35°C were 13.5- and 5.04- higher, respectively, than trehalose levels in cells grown in minimal medium without NaCl added. These data suggest that, although temperature stress alone induces some trehalose synthesis by *R. etli*, trehalose biosynthesis in this microorganism is mainly triggered by osmotic stress. 

### Genomic analysis of trehalose metabolism in *R. etli*

**Figure 2:**
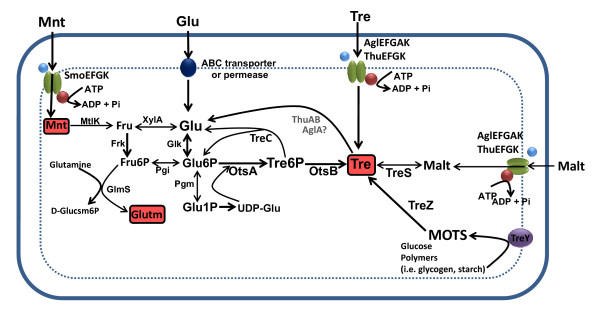
**Scheme of trehalose metabolism in *R. etli* based on the annotated genome.** Abbreviations used: Glu, D-glucose; Glu6P, D-glucose-6-phosphate; Glu1P, D-glucose-1-phosphate; Glutm, D-Glutamate, D-Glucsm6P, D-Glucosamine-6-phosphate; Fru, D-fructose; Fru6P, D-fructose-6-phosphate; Malt, Maltose; Mnt, mannitol, MOTS, Maltoolygosyltrehalose; Tre, Trehalose; TreP, Trehalose-6-phosphate; AlgEFGAK and ThuEFGK, putative Trehalose/maltose/sucrose ABC transporters; GlmS, glucosamine-6-phosphate synthase; Mtlk, Mannitol 2-dehydrogenase; Frk, Fructokinase, OtsA, Trehalose-6-phosphate synthase, OtsB, Trehalose-6-phosphate phosphatase; Pgi, Phosphoglucose isomerase; XylA, Xylose isomerase; TreC, Trehalose-6-phosphate hydrolase; TreS, Trehalose synthase; TreY, Maltooligosyl trehalose synthase; TreZ, Maltooligosyl trehalose trehalohydrolase, SmoEFGK*,* Sorbitol/mannitol ABC transporter.

Trehalose synthesis and catabolism have proven to be relevant for the symbiotic performance of rhizobia [5,10,21,22]. To get an overview of the metabolism of trehalose in *R. etli*, we inspected its genome for genes involved in trehalose synthesis, transport and degradation. Genes for trehalose metabolism were scattered among the chromosome and plasmids a, c, e, and f (see Additional file 1: Table S1 and Additional file 2: Figure S1A, for a complete description of gene annotation and gene clustering). As suggested by Suarez et al. [10] putative genes encoding the three so far known trehalose synthesis pathways in rhizobia (TreYZ, TreS and OtsAB) are present in *R. etli*. First, genes encoding trehalose synthesis from glucose polymers were found in plasmid p42e (*treY*)*,* and the chromosome and plasmid p42f (two copies of *treZ*). Second, two genes encoding a putative trehalose synthase (TreS) were found in the chromosome and plasmid p42f. The product of the chromosomal putative *treS* gene presented similar length and significant sequence identity to known trehalose synthases from bacteria, but the product of the plasmid f-borne *treS*-like gene carried an additional domain of unknown function (DUF3459).Third, two genes were annotated as *otsA*, one located in the chromosome (*otsAch*) and one in plasmid p42a (*otsAa*). Both products showed homology to trehalose 6-phosphate synthases from other rhizobia, but the identity was much higher for OtsAch. In addition, a gene annotated as *otsB* was located in plasmid c. As trehalose is synthesized by *R. etli* from mannitol (see Figure 1), we searched for genes involved in mannitol transport and conversion into glucose. The genome of *R. etli* does not encode a specific mannitol phosphotransferase, suggesting that mannitol does not use this system to enter the cell. Instead, we found *smoEFGK* (encoding a sorbitol/mannitol ABC transporter), *mtlK* (encoding a mannitol 2-dehydrogenase that converts mannitol to fructose), and *xylA* (encoding a xylose isomerase that converts fructose to glucose. All these findings suggest that *R. etli* can convert mannitol into glucose via fructose. *R. etli* CE3 grown in minimal medium B^-^ also accumulates glutamate (see below). Since B^-^ does not contain ammonium, the most plausible route for glutamate biosynthesis from mannitol is through the enzyme glucosamine-6-phosphate synthase, which converts D-fructose-6-phosphate and L-glutamine into D-glucosamine-6-phosphate and L-glutamate. Two copies of the encoding gene (*glmS*) were found in *R. etli* chromosome (Additional file 1: Table S1, Figure 2). A previous study suggested that *R. etli* can degrade trehalose [53]. Therefore, we also looked for genes involved in uptake and degradation of trehalose. We did not find a trehalose phosphotransferase system (PTS) for the uptake of trehalose, but two putative ABC uptake systems homologous to those operating in *S. meliloti*[22,23] were found that might be involved in the uptake of trehalose, sucrose, and/or maltose. These were encoded in plasmid p42f (ThuEFGK), and the chromosome (AglEFGK). Regarding trehalose degradation, neither *E. coli treA*- or *treF-* like genes for periplasmic or cytoplasmic trehalases, respectively, nor genes belonging to glycoside hydrolase family 15 trehalases [16,17], were found in the *R. etli* genome. However, orthologs to the *thuAB* genes, which encode the major pathway for trehalose catabolism in *S. meliloti*[21], were found in the chromosome and plasmid p42f. In addition, three copies of *treC*, encoding putative trehalose-6-phosphate hydrolases, were identified in the chromosome. All three TreC proteins belonged to the family 13 of glycoside hydrolases [16], but they did not cluster together (see the phylogenetic tree in Additional file 2: Figure S1B). The metabolism of trehalose in *R. etli* inferred from its genome sequence is summarized in Figure 2. 

### Phylogenetic analysis of the two *R. etli* trehalose-6-phosphate synthases

**Figure 3:**
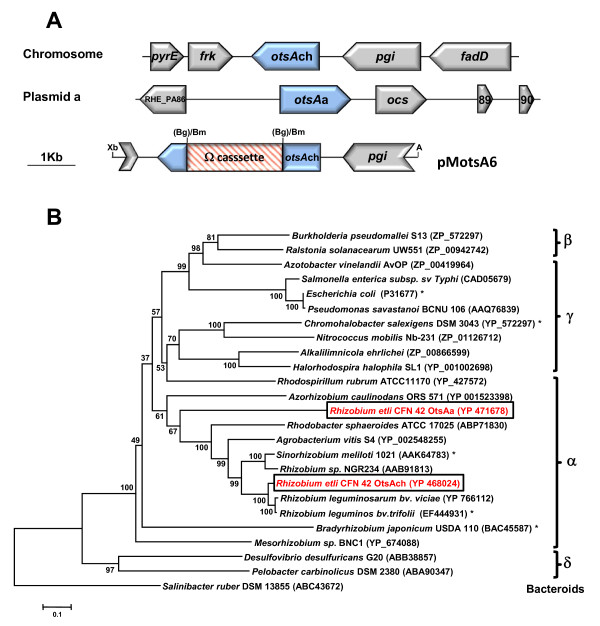
***In silico* analysis of the two trehalose-6-phosphate synthases (OtsA) encoded by the *R. etli* genome.** (**A**) Genomic context of the *otsAch* (chromosomal) and *otsAs* (plasmid p42a) genes, and construction of an *otsAch* mutant. *otsAch* was inactivated by the insertion of a *Bam*HI (Bm)-digested Ω cassette, which carried resistance genes for streptomycin/spectinomycin, into its unique site *Bgl*II (Bg), giving the plasmid pMotsA6 (see text for details). (**B**) Neighbor-joining tree based on OtsA proteins from α-, β-,γ and δ-proteobacteria. The tree is drawn to scale, with branch lengths in the same units as those of the evolutionary distances used to infer the phylogenetic tree. The Bacteroides/Chlorobi representative *S. ruber* was used as outgroup. The evolutionary distances were computed using the Poisson correction method and are in the units of the number of amino acid substitutions per site. The rate variation among sites was modeled with a gamma distribution (shape parameter = 1). All positions containing gaps and missing data were eliminated from the dataset (complete deletion option). Bootstrap probabilities (as percentage) were determined from 1000 resamplings.

As two copies of OtsA (OtsAch and OtsAa, Figure 3A) were encoded by the *R. etli* genome, we investigated their phylogenetic relationship. First we aligned the amino acid sequences of both *R. etli* OtsA proteins with the sequences of characterized trehalose-6-P- synthases, and compared motifs involved in enzyme activity. All residues corresponding to the active site determined in the best studied *E. coli* trehalose-6-P synthase [54] were conserved in *R. etli* OtsAch and OtsAa (data not shown). However, the identity between both proteins was only of 48%, and the gene *otsAa* was flanked by putative insertion sequences in the *R. etli* genome. In addition, the *otsAch* copy and *R. etli* genome had a similar codon use, whereas the *otsAa* copy showed a different preference for Stop codon, and codons for amino acids as Ala, Arg, Gln, Ile,Leu, Phe, Ser, Thr, and Val. These findings suggested that *otsAa* might have been acquired by horizontal transfer. To check this hypothesis, we constructed a phylogenetic tree with sequences of proteobacterial OtsA proteins available in the data bases, including OtsAs from *R. etli* and other rhizobia like *R. leguminosarum* bv. *trifolii*[7], *S.meliloti*[5], and *B. japonicum*[2]. As shown in Figure 3B, the resulting phylogenetic tree showed four separated branches, with a generally homogeneous distribution of phylogenetic groups. The first branch was formed by OtsA proteins from β- and γ -proteobacteria, including OtsA from *E. coli* and *Salmonella enterica*. The second cluster was mainly composed of OtsA proteins from γ-proteobacteria, including some halophilic representatives such as *C. salexigens* and *Halorhodospira halophila*. The third branch grouped OtsAs from α-proteobacteria, including the two *R. etli* OtsA proteins. Whereas *R. etli* OtsAch grouped with OtsAs from *R. leguminosarum, S. meliloti, Rhizobium* sp. NGR234 and *Agrobacterium vitis*, *R. etli* OtsAa constituted a separated sub-group within the α-proteobacterial branch. The fourth branch was composed by OtsA proteins from δ-protobacteria. Some incongruences were found, as *B. japonicum* and *Mesorhizobium* proteins did not clustered with OtsA proteins from other rhizobia. In summary, this phylogenetic analysis supports the hypothesis that *otsAa* was transferred to *R. etli* or its ancestor from a related α-proteobacteria, which did not belong to the *Rhizobium*/*Agrobacterium* group. 

### Inactivation of *R. etli otsA*ch totally suppresses trehalose synthesis from mannitol

**Figure 4:**
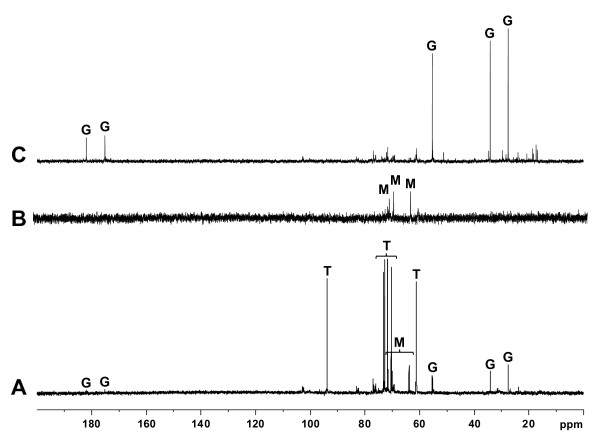
**Natural abundance **^**13**^** C-NMR spectrum of major cytosolic solutes accumulated by *R. etli* wild-type and *otsAch* strains.** Wild-type (**A**) and *otsAch* (**B**, **C**) cells were grown at 28°C in B^-^ minimal medium with 0.2 M NaCl. Cells were extracted as described in Materials and Methods. For the *otsAch* strain, cells were collected at the entrance of the first (**B**) and second (**C**) stationary phase of growth. The major solutes were trehalose (T), glutamate (G) and mannitol (M).

From the above phylogenetic analysis, *otsAch* was chosen as the most promising candidate to encode a functional trehalose-6-P-synthase. To check this, the corresponding mutant (strain CMS310) was constructed by insertion of an omega cassette within *otsAch* (Figure 3A), followed by double recombination in the chromosome of the wild-type strain. Then, we used natural abundance ^13^C-NMR to determine the compatible solute pool of wild type and *otsAch* cells grown at 28°C in 0.2 M NaCl B^-^ medium up to early-stationary phase. As shown in Figure 4A, the ^13^C-NMR spectrum of *R. etli* wild-type strain contained three sets of chemical shifts, which were assigned to trehalose (61.2, 70.4, 71.7, 72.8, 73.2 and 93.9 ppm), mannitol (63.9, 70.0 and 71.6 ppm) and glutamate (27.6, 34.2, 55.4, 175.2 and 181.9 ppm). Although^13^C-NMR is only a semi-quantitative technique, it was evident that trehalose levels were much higher than those of mannitol and glutamate, suggesting that trehalose is the major compatible solute of *R. etli* under these conditions. Mannitol was absent when glucose was used as a sole carbon source (data not shown), indicating that it was accumulated by *R. etli* after its uptake from the external medium. Chemical shifts corresponding to trehalose were not present in the spectrum of the *R. etli otsA*ch strain, where only signals corresponding to mannitol were detected (Figure 4B). From these results, we conclude that the product encoded by *otsA*ch is involved in trehalose synthesis in *R. etli.* Moreover, at least under the conditions tested, the *otsAa* copy does not seem to be functional.

### Trehalose synthesis mediated by *otsAch* is essential for thermoprotection of *R. etli*

**Figure 5:**
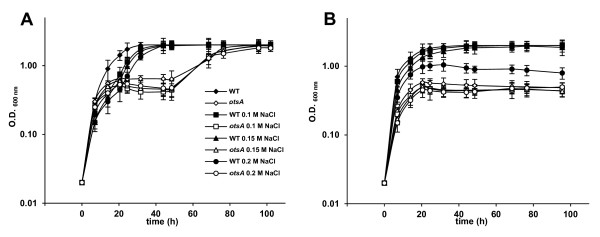
**Contribution of trehalose to salinity and heat tolerance of *R. etli.*** Cells of *R. etli* wild-type (black markers) and *otsAch* mutant (white markers) were grown in minimal medium B^-^ with 0.0 or 0.2 M NaCl at 28°C (**A**) or 35°C (**B**). 10 g l^-1^ mannitol was used as the sole carbon source. Values shown are the mean of two replicas of each condition in three independent experiments ± SD (standard deviation).

We investigated the effect of a mutation in *otsAch* on *R. etli* heat tolerance. For this purpose, we compared the growth of wild-type and *otsA*ch strains in minimal medium B^-^ under different combinations of osmotic (0.0 M to 0.2 M) and heat (28°C or 35°C) stresses. As previously described (see Figure 1), at optimal temperature (28°C), the wild-type strain grew optimally without NaCl added. At higher salinities (0.1 to 0.2 M NaCl), wild-type cells showed a delayed growth, but eventually they reached a stationary phase with absorbance values comparable to those of cultures without NaCl (Figure 5A).

Interestingly, *otsAch* cells grown at 28°C with mannitol showed a biphasic pattern, which was independent of the NaCl concentration, although cells exposed to osmotic stress were more affected than cells grown without NaCl. The first stage consisted of an attenuated exponential phase of 20 h (if compared with that of the wild type) followed by 30 h of arrested growth with OD_600_ values of around 0.5 units. In the second one, growth was restarted, showing a second exponential phase during 40 h, followed by a second stationary phase with absorbance values comparable to those of the wild type strain (Figure 5A). As in *otsAch* cells collected at the beginning of the first stationary phase (see Figure 4B), trehalose was absent from extracts prepared from samples harvested at the entrance of this second stationary phase. Instead, they contained large amounts of glutamate (Figure 4C). However, when glucose and trehalose were used as the sole carbon source, this biphasic pattern of growth was not observed. Growth of the *otsAch* strain with both carbon sources was delayed with respect to the wild-type strain, even in the absence of osmotic stress (see Additional file 3: Figure S2).

At 35°C, *R. etli* wild-type strain was able to grow well in B^-^ medium with NaCl concentrations ranging from 0 to 0.15 M. As described above (see Figure 1), growth of the wild type was impaired at 35°C with 0.2 M NaCl, showing absorbance values not exceeding 1.0 unit of OD_600_(Figure 5B). At this temperature, growth of the *otsA* mutant was severely affected, regardless of the salinity of the culture medium, with cultures showing OD_600_ around 0.5 OD units. The above data suggest that trehalose is essential for growth of *R. etli* at high temperature.

### Osmotically induced trehalose synthesis improves desiccation tolerance in *R. etli*

Involvement of trehalose in desiccation tolerance in rhizobia has been firmly established in *R. leguminosarum* bv. *trifolii*[7]. On the other hand, in *S. meliloti*[55] or rhizobia nodulating *Acacia*[56], desiccation tolerance was stimulated by osmotic and/or temperature pre-treatment. To check the influence of trehalose on desiccation tolerance of *R. etli*, wild type and *otsAch* strains were grown at 28°C in minimal medium B^-^alone or additioned with 0.2 M NaCl, and harvested at early stationary phase. For cell drying, we used two variants of the protocol described by Manzanera et al. for *E. coli*[39], a drying process (induced by vacuum at 30°C) or a drying + high temperature process (including a second step with a controlled increase of temperature from 20 to 30°C under vacuum).

**Figure 6:**
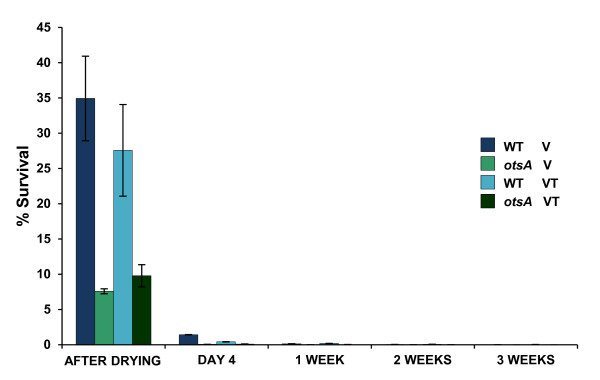
**Survival of *R. etli strains* after vacuum-drying and subsequent storage at 28°C.***R. etli* wild-type and *otsAch* mutant were cultured at 28°C in minimal medium B^-^ with 0.2 M NaCl until they reached early stationary phase: Desiccation was performed as described in Methods, using vacuum or vacuum + temperature conditions. After drying, samples were sealed and stored at 28°C. Viability was measured before (taken as 100% survival), just after drying, and after 4 days, 1, 2 and 3 weeks storage, and expressed as percentage of viable cells. Error bars indicate standard deviations.

In the absence of osmotic stress, both wild type and *otsAch* strains showed survival levels under 0.01%, regardless of the drying protocol (data not shown). In contrast, wild type cells osmotically pre-conditioned by the presence of 0.2 M NaCl showed ca. 35% survival levels after drying, although viability after 4 days storage dropped down to 1.4% (Figure 6). Compared to the drying treatment, the drying + high temperature protocol did not enhance wild type cell survival (Figure 6). The same drying experiments were performed with trehalose-deficient (*otsAch*) cells harvested at early stationary phase. Regardless of the protocol used, the *otsAch* strain showed ca 3-fold lower survival levels than the wild type strain after the drying process, and a null viability after 4 days storage. These findings suggested (i) a beneficial effect of osmotic stress in *R. etli* tolerance to desiccation, and (ii) a role of trehalose on desiccation tolerance in *R. etli*.

### Symbiotic phenotype of the *R. etli otsAch* mutant

To analyze if the *otsAch* mutation modifies the capacity of *R. etli* to fix nitrogen in symbiosis, common bean plants were inoculated with *R. etli* wild-type and the *otsAch* strain. After inoculation, plants were grown under optimal (control plants) or water deficit conditions and were evaluated for nodulation, plant dry weight, total nitrogen content, nitrogenase activity, and leghaemoglobin content of the nodules. Plant water status during the different treatments was monitored by measuring water potential (Ψ_w_) of the first fully expanded leaf. Water potential in plants subjected to drought stress by holding irrigation for 5 days reached values of about −1 ± 0.25 MPa (moderate drought). When irrigation was stop for 10 days, leaf Ψ_w_ reached values of about −2 ± 0.3 MPa (severe drought). The control plants maintained a leaf Ψw of −1 ± 0.4 MPa. The effect of either moderate or severe drought stress in leaf Ψw of plants inoculated with the *otsAch* mutant was similar to that of plants inoculated with the wild type (data not shown).

Independently of the plant treatment, no significant differences were observed in nodulation, plant growth parameters, and nitrogen fixation parameters among plants inoculated with any of the strains (Table 2). A moderate drought did not affect nodules number (NN), nodule dry weight (NDW), plant dry weight (PDW), and total nitrogen content (TN) of plants inoculated with either the wild-type or the *otsAch* strain (Table 2). Specific nitrogenase activity expressed as acetylene reduction activity (ARA) and leghaemoglobin (Lb) content of the nodules as an estimation of nodule functionality were also measured. Regardless of the plant treatment, inoculation of plants with the *otsAch* mutant did not affect significantly ARA or Lb content compared to those plants inoculated with the wild-type strain (Table 2).

**Table 2:** **Symbiotic phenotype of the*otsAch*mutant**

		**Moderate drought**								**Severe drought**							
**Strain**	**Treatment**																
		**NN**	**NDW**	**PDW**	**TN**	**ARA**	**Lb**	**Tre (B)**	**Tre (C)**	**NN**	**NDW**	**PDW**	**TN**	**ARA**	**Lb**	**Tre (B)**	**Tre (C)**
**Wild-type**	Control	48 ± 6a	23 ± 3a	0.31 ± 0.02a	4.5 ± 0.3	24 ± 1a	33 ± 1a	1.17 ± 0.32a	ND	56 ± 4a	35 ± 3a	0.51 ± 0.03a	12 ± 0.4a	22 ± 0.6a	37 ± 2a	2.12 ± 0.43b	ND
	Stress	45 ± 4a	22 ± 3a	0.34 ± 0.02a	5.0 ± 0.2	22 ± 2a	32 ± 1a	1.24 ± 0.20a	ND	31 ± 3b	16 ± 1b	0.34 ± 0.03b	5.3 ± 0.1b	14 ± 0.7b	24 ± 1b	2.37 ± 0.39b	ND
***otsAch***	Control	48 ± 5a	24 ± 3a	0.37 ± 0.03a	5.5 ± 0.4	27 ± 3a	35 ± 3a	1.15 ± 0.29a	ND	61 ± 4a	42 ± 5a	0.52 ± 0.03a	12.5 ± 0.5a	27 ± 1.2a	41 ± 4a	1.90 ± 0.32b	ND
	Stress	46 ± 5a	25 ± 5a	0.35 ± 0.05a	5.3 ± 0.3	24 ± 1a	35 ± 3a	1.25 ± 0.30a	ND	35 ± 5b	19 ± 3b	0.37 ± 0.03b	5.5 ± 0.3b	16 ± 1.5b	25 ± 1b	2.08 ± 0.37b	ND

Nodules number (NN), nodule dry weight [NDW, (mg plant^-1^)], plant dry weight [PDW, (g plant^-1^)], total nitrogen content [TN, (mg plant^-1^)], acetylene reduction activity [ARA, (μmol C_2_H_4_ h^-1^ g^-1^ NDW)],leghaemoglobin [Lb, (mg Lb g^-1^ NDW)], and trehalose (Tre) in bacteroids (B) and nodule cytosol (C) [μmol gDW^-1^] content in nodules and plants subjected or not (control) to moderate or severe drought conditions. Values in a column followed by the same lower-case letter are not significantly different as determined by the Tukey HSD test at *P* ≤ 0.05 (n = 9).

ND. Not detected.

As shown in Table 2, NN and NDW per plant was negatively affected by a severe drought since a decrease of about 45% and 53% in those parameters was observed in plants inoculated with the wild-type strain compared to control plants. A similar decrease of NN (43%) and NDW (49%) was observed in plants subjected to a severe stress and inoculated with the *otsAch* mutant compared to control plants (Table 2). After a severe drought, a 53% and 49% reduction of PDW was observed in plants inoculated with the wild-type or the *otsAch* mutant, respectively. Plants inoculated with any of the strains and subjected to severe drought showed a similar reduction of about 30% in TN compared to control plants (Table 2). Plants inoculated with the wild-type strain and subjected to severe drought showed an inhibition of ARA of about 36% compared to control plants. This activity was similarly dropped in nodules produced by the *otsAch* mutant under severe drought (41% compared to control plants) (Table 2). A severe drought provoked a significant decline in Lb content of about 35% in plants inoculated with the wild-type strain compared to control plants Likewise, this parameter was also reduced of about 39% in plants inoculated with the *otsAch* mutant and subjected to a severe drought (Table 2). Finally, trehalose content in bacteroids of the wild type and *otsAch* strains was similar, regardless of the treatment, suggesting that under symbiotic conditions (i.e. with other trehalose precursors available) other trehalose synthesis genes (i.e. TreS or TreYZ) may be operating. Trehalose was not detected in the cytosol of nodules induced by either the wild type or the *otsAch* strain under any condition tested, suggesting that trehalose in the *R. etli*-induced nodules is synthesized only by the microsymbiont.

## Discussion

Trehalose in rhizobia is a key compound for signaling plant growth, yield and adaptation to abiotic stress, and its manipulation has a major agronomical impact on leguminous plant. In this work we reconstructed trehalose metabolism in *R. etli*, and investigated the role of trehalose in the response to high temperature and desiccation stress, as well as symbiotic performance. By using^13^C-NMR, we showed that besides trehalose as the major compatible solute, *R. etli* CE3 also amasses glutamate. In addition, it can accumulate mannitol if present in the external medium. The same compatible solute profile was recently reported for the strain *R. etli* 12a3, isolated from *P. vulgaris* nodules in Tunisian fields [6].

Two successive genome-based metabolic reconstructions of *R. etli* have been reported, covering in total 405 reactions and 450 (but not trehalose-related) genes [57,58]. In this study, we reconstructed the metabolism of trehalose in *R. etli*, including trehalose uptake, degradation, and synthesis (see Figure 2). Our data suggest that uptake and catabolism of trehalose in *R. etli* uses the same pathways as in *S. meliloti*, since orthologs to the *S. meliloti* AglEFGK/ThuEFGK ABC trehalose/maltose/sucrose transporters [22,23], as well as the ThuAB catabolic route [21], were found in *R. etli*. In addition, *R. etli* genome accounts for up to 3 putative copies of the trehalose-6-phosphate hydrolase (TreC). Only TreC3 was in the same group as the characterized TreC protein from *E. coli*, suggesting that the other copies might have a slightly different function. Interestingly, *treC2* (annotated as *aglA*) was located upstream of the *aglEFGK* genes encoding the alpha-glucoside ABC transporter. In *S. meliloti, aglA*, encoding an alpha-glucosidase with homology to family 13 of glycosyl hydrolases, forms part of the *aglEFGAK* operon, suggesting a possible function in sucrose, maltose and/or trehalose catabolism. Further work is necessary to elucidate the role of the different systems involved in trehalose transport and degradation in *R. etli.*

Regarding trehalose synthesis, Suarez et al. [10] already suggested the presence in *R. etli* of the three trehalose biosynthetic pathways so far known in rhizobia (OtsAB, TreS, and TreYZ). In this work, we precisely located the corresponding genes, and proposed the most plausible route of glucose synthesis from mannitol, and subsequent OtsAB-mediated trehalose synthesis (see Figure 2).

We found that genes for trehalose metabolism were scattered in the genome, and sometimes present in more than one copy (i.e., *otsA*, *treZ*, *treS*, *treC*). This high enzyme redundancy seems to be a general characteristic of *R. etli* CFN 42, and was proposed to correlate with the different degrees of metabolic responses and alternative regulation necessary to cope with a challenging environment without compromising the integrity of the pathways [30]. Despite the active site residues were conserved in the p42a-encoded copy of OtsA, our phylogenetic analysis, together with the presence of insertion sequences flanking the gene, and its different codon used (if compared to the *R. etli* chromosome), strongly suggests that *otsAa* was acquired by lateral transfer. All these findings agree with the proposal by González et al. [30] about an exogenous origin for *R etli* p42a.

The role of trehalose in the osmostress response has been widely demonstrated in many bacteria, including *S. meliloti*[5], *B. japonicum*[2] and *R. etli*[10]. In the former species, the involvement of trehalose in osmoadaptation was proposed based on three findings: (i) trehalose accumulation in the wild type was osmoregulated, (ii) an *otsA* mutant was osmosensitive, and (iii) overexpression of *otsA* led to an increased osmotolerance. Our results confirm the previous result that trehalose biosynthesis in *R. etli* is triggered by osmotic stress. However, the *otsAch* mutant reported in this work was much less affected by NaCl stress than the *otsA* mutant described by Suarez et al. [10]. These authors tested osmosensitivity in a glycerol minimal medium with 0.5 M NaCl during 48 h. In contrast, we found that the *R. etli* wild type strain could not grow above 0.2 M NaCl in B^-^ mannitol minimal medium. Therefore, it is possible that the *otsAch* mutant described here might show an increased osmosensitivity at higher salinities. On the other hand mannitol, which was accumulated as an osmoprotectant (see Figure 4A), might have partially restored the growth of the *otsAch* strain when it was used as a carbon source.

Notably, extracts of *otsAch* cells grown with mannitol contained large amounts of glutamate, which was the predominant compatible solute (see Figure 4C). Thus, glutamate seems to be important for the long term adaptation of *R. etli* to osmotic stress, at least in the *otsAch* mutant strain describe here. Very interestingly, growth of the *otsAch* mutant was also affected in the absence of salinity stress (see Figure 5 and Additional file 3: Figure S2), suggesting an important role of trehalose in *R. etli* physiology. Trehalose has been described to be essential as cell wall and membrane precursor [59], as membrane stabilizer [60], or as antoxidant [61], to give some examples. This apparent essentiality of trehalose for normal growth of *R. etli* deserves further investigation.

A high level of trehalose accumulation is an important factor in the heat shock response in yeast [25]. In addition, bacteria such as *E. coli* and *S. enterica* serovar Typhimurium accumulate trehalose in response to heat stresses, and transcription of the *otsAB* genes for trehalose synthesis is thermoregulated [27,62]. In this work, we show the relevance of trehalose for *R etli* tolerance to high temperature. Although, trehalose content in *R. etli* cells grown at high temperature was very low, these levels were apparently enough as to protect wild type cells against heat stress, as growth of the trehalose deficient *otsAch* strain was impaired at high temperature. Similarly in *E. coli,* stationary phase induced thermotolerance has been shown to depend upon the *rpoS* regulated expression of the *otsAB* genes for trehalose synthesis, but the levels of trehalose synthesized on entry into stationary phase were very much lower than in osmotically stressed cells [26]. There is now a large body of evidence showing that the mechanisms for trehalose-mediated protection against heat and desiccation stress are different from those involved in osmoprotection, i.e., as a counteracting osmolyte. Thus, studies *in vitro* have shown that trehalose preserves structure and function in biomolecules and molecular assemblages, such as membranes, during drying and heat stress [63].

Strains of *R. leguminosarum* bv *trifolii*[7] and *R. etli* (this work) deficient in trehalose synthesis are more sensitive to the effects of drying, and show impaired survival upon storage. Thus, desiccation tolerance in *R. etli* cells was dependent of high trehalose production by osmotic pre-conditioned cells. Indeed, desiccation stress is much more harmful than heat stress for microorganisms, as it produces the accumulation of salt and solutes, hyperosmotic stress, metabolism impairment, and damage to macromolecules upon removing the aqueous monolayer [64]. This may explain why high trehalose content is necessary for survival of *R. etli* cells to drying*,* in order to cope with so many stresses. In agreement with this, *E. coli*[65], *S. meliloti*[55], and desert-isolated rhizobial strains nodulating acacia [56] that were osmotically induced to accumulate trehalose (and also mannosucrose, in desert-isolated rhizobia), showed increased tolerance to drying and storage. Interestingly, transcriptomic analyses revealed that desiccation stress *per se,* if performed under controlled conditions, also induced trehalose synthesis by *B. japonicum*[24], the soil actinomycete *Rhodococcus jostii*[66] and the yeast *Saccharomyces cerevisiae*[67].

It is worth mentioning that desiccation tolerance by *R. etli* was not improved by an increase in drying temperature. This lack of correlation has been also found in many other rhizobia [64] and could be attributed, at least in *R. etli*, to the low induction of trehalose synthesis under high temperature. On the other hand, the survival rate of *R*. *etli* wild type strain after the vacuum-drying treatments was below 40%, and rapidly decreased after 4 days storage (see Figure 6). This differs from the high survival rates found for *S. meliloti* on nitrocellulose filters [55] or *R. leguminosarum* bv *trifolii* on glass beads [7]. Rather than intrinsic tolerance to desiccation, we suggest that these differences may be related to the experimental conditions used for drying.

In rhizobia, the relationship between inactivation of a given trehalose metabolic pathway (and the resulting trehalose accumulation) and the observed symbiotic performance, seems to vary among species (see Introduction). The *R. etli otsA* mutant reported by Suarez et al. [10] was affected in its capacity to establish an efficient symbiosis with bean plants. However, bacteroids of the *R. etli otsAch* mutant constructed in this work showed the same trehalose levels than those of the wild type, and were not affected in its symbiotic performance. The reasons for these differences remain to be elucidated, but it is plausible that under the conditions used in our symbiosis experiments other trehalose synthesis pathways were activated in the *otsAch* strain, including the *otsAa* copy, that may compensate the lack of *otsAch*. Thus, our results do not preclude a role of trehalose in the *R. etliPhaseolus vulgaris* symbiosis.

In its natural habitat, soil bacteria as *R. etli* are subjected to fluctuating osmotic, temperature and desiccation constrains. Improving trehalose production in *R etli* has been shown to be a useful strategy to achieve drought tolerance of the bean plant host [10]. In this work, we have shown that trehalose is essential for *R. etli* survival to high temperature and drying under free living conditions. Thus, engineering trehalose accumulation promises to be useful to improve survival of *R. etli*-based inoculants during desiccation stress in storage, upon application to seeds, or once released in fields.

## Conclusions

In bacteria, hyperosmotic, heat and drought stresses involve a number of multiple and complex responses, which in some cases are interrelated. Desiccation tolerance is special, as any response against this stress should be sensed and elicited before the water activity is too low as to respond to. In *B. japonicum*, controlled desiccation conditions resulted in a significant induction of the *otsA*, *otsB* and *treS* genes for trehalose synthesis, as well as increased trehalose levels. However, in Nature drying may be so rapid as to preclude any metabolic response. Thus, it is reasonable to assume that desiccation tolerance may be either a constitutive trait or conditioned to the responses to other stresses such as high salinity, heat, or oxygen stress. In the example illustrated in this work, the disaccharide trehalose was involved in the *R. etli* response to the three stresses, suggesting that it is a common element of the general abiotic stress response of this microorganism. One of the most interesting findings of this study was that high temperature did not induce a dramatic accumulation of trehalose by *R. etli*, although trehalose levels were enough as to cope with high temperature. Thus, our results suggest that selection of heat tolerant strains might not always ensure a concomitant enhanced drought tolerance, at least if the strategy is based upon a higher trehalose accumulation. On the other hand, desiccation seems to be the most deleterious stress for *R. etli*, and apparently demanded a higher, osmotic stress-dependent, trehalose production in order to survive.

## Authors’ contributions

MRB and MA performed the majority of the experiments, participated in bioinformatics analysis, study design, and in crafting of the manuscript. AH, MJD and FIG performed symbiosis experiments and RMN analyses. JJN and CV conceived the study, participated in the design, coordination, bioinformatic analysis, and crafting of the manuscript. All authors have read and approved the final manuscript.

## Supplementary Material

Additional file 1 **Table S1. **R. *etli* genes involved in trehalose and glutamate metabolis. Click here for file

Additional file 2 **Figure S1. **Genomic analysis of R. *etli* pathways involved in trehalose metabolism. (A) Genomic context of genes involved in trehalose metabolism. Position and clustering of genes included in Additional file 1: Table S1. are indicated. (B) Neighbor-joining tree based on proteins belonging to families 13 and 15 of glycosydases, including the three TreC-like proteins from *R. etli*. The tree is drawn to scale, with branch lengths in the same units as those of the evolutionary distances used to infer the phylogenetic tree. The *E. coli* and *Rhrodothermus marinus* representatives were used as outgroup. The evolutionary distances were computed using the Poisson correction method and are in the units of the number of amino acid substitutions per site. The rate variation among sites was modeled with a gamma distribution (shape parameter = 1). All positions containing gaps and missing data were eliminated from the dataset (complete deletion option). Bootstrap probabilities (as percentage) were determined from 1000 resamplings. Click here for file

Additional file 3 **Figure S2.** Growth of R. wild type (WT) and the otsAch mutant CMS310 with trehalose and glucose as the sole carbon source. Cells were grown in at 28°C in B- minimal medium with 20 mM trehalose or glucose and 0.0 or 0.2 M NaCl. Click here for file
