# Supplementary material for: Role of trehalose in heat and desiccation tolerance in the soil bacterium Rhizobium etli
Source: BMC Microbiol. 2012 Sep 17;12:207. doi: 10.1186/1471-2180-12-207 (PMC3518184; doi:10.1186/1471-2180-12-207)
Supplement: Additional file 1 — Table S1. R. etli genes involved in trehalose and glutamate metabolis. [file 1471-2180-12-207-S1.pdf]

Table S1. *R. etli* genes involved in trehalose and glutamate metabolism

gene	Protein	chromosome	plasmid a	plasmid c	plasmid e	plasmid f
**Trehalose synthesis**						
otsA	trehalose 6-phosphate synthase	**RHE_CH00476**	**RHE_PA00087**			
otsB	trehalose-6-phosphate phosphatase			**RHE_PC00228**		
treS	trehalose synthase	**RHE_CH02260**				**RHE_PF00276**
treZ	maltooligosyl-trehalose trehalohydrolase	**RHE_CH03279**				**RHE_PF00263**
treY	maltooligosyl-trehalose synthase				**RHE_PE00008**	
**Trehalose/maltose uptake**						
thuE	trehalose/maltose ABC transporter, sugar-binding protein					**RHE_PF00210**
thuF	trehalose/maltose ABC transporter, permease protein					**RHE_PF00209**
thuG	trehalose/maltose ABC transporter, permease protein					**RHE_PF00208**
thuK	trehalose/maltose ABC transporter, ATP-binding protein					**RHE_PF00207**
aglE	alpha-glucoside ABC transporter, substrate-binding protein	**RHE_CH00696**				
aglF	alpha-glucoside ABC transporter, permease protein	**RHE_CH00697**				
aglG	alpha-glucoside ABC transporter, permease protein	**RHE_CH00698**				
aglK	alpha-glucoside ABC transporter, ATP-binding protein	**RHE_CH00700**				
**Trehalose degradation**						
thuA		**RHE_CH03256**				**RHE_PF00206**
thuB		**RHE_CH03255**				**RHE_PF00205**
treC1		**RHE_CH03095**				
treC2 (aglA)		**RHE_CH00699**				
treC3(malL)		**RHE_CH03282**				
**Mannitol/glucose metabolism**						
smoE	sorbitol/mannitol ABC transporter, substrate-binding protein	**RHE_CH03682**		**RHE_PC00219**		
smoF	sorbitol/mannitol ABC transporter, permease protein	**RHE_CH03681**				
smoG	sorbitol/mannitol ABC transporter, permease protein	**RHE_CH03680**				
smoK	sorbitol/mannitol ABC transporter, ATP-binding protein	**RHE_CH03679**				
mtlK	mannitol 2-dehydrogenase	**RHE_CH03678**				
xylA	xylose isomerase	**RHE_CH03648**				
frK	fuctokinase	**RHE_CH00475**				
glk	glucokinase	**RHE_CH00172**				
pgi	glucose-6-phosphate isomerase	**RHE_CH00477**				
pgm	phosphoglucomutase	**RHE_CH03597**				
**Glutamate metabolism**						
glmS1	glucosamine-6-phosphate synthase	**RHE_CH02091**				
glmS1	glucosamine-6-phosphate synthase	**RHE_CH04036**				
