# Supplementary material for: Role of trehalose in heat and desiccation tolerance in the soil bacterium Rhizobium etli
Source: BMC Microbiol. 2012 Sep 17;12:207. doi: 10.1186/1471-2180-12-207 (PMC3518184; doi:10.1186/1471-2180-12-207)
Supplement: Additional file 2 — Figure S1. Genomic analysis of R. etli pathways involved in trehalose metabolism. (A) Genomic context of genes involved in trehalose metabolism. Position and clustering of genes included in Additional file 1: Table S1. are indicated. (B) Neighbor-joining tree based on proteins belonging to families 13 and 15 of glycosydases, including the three TreC-like proteins from R. etli. The tree is drawn to scale, with branch lengths in the same units as those of the evolutionary distances used to infer the phylogenetic tree. The E. coli and Rhrodothermus marinus representatives were used as outgroup. The evolutionary distances were computed using the Poisson correction method and are in the units of the number of amino acid substitutions per site. The rate variation among sites was modeled with a gamma distribution (shape parameter = 1). All positions containing gaps and missing data were eliminated from the dataset (complete deletion option). Bootstrap probabilities (as percentage) were determined from 1000 resamplings. [file 1471-2180-12-207-S2.pdf]

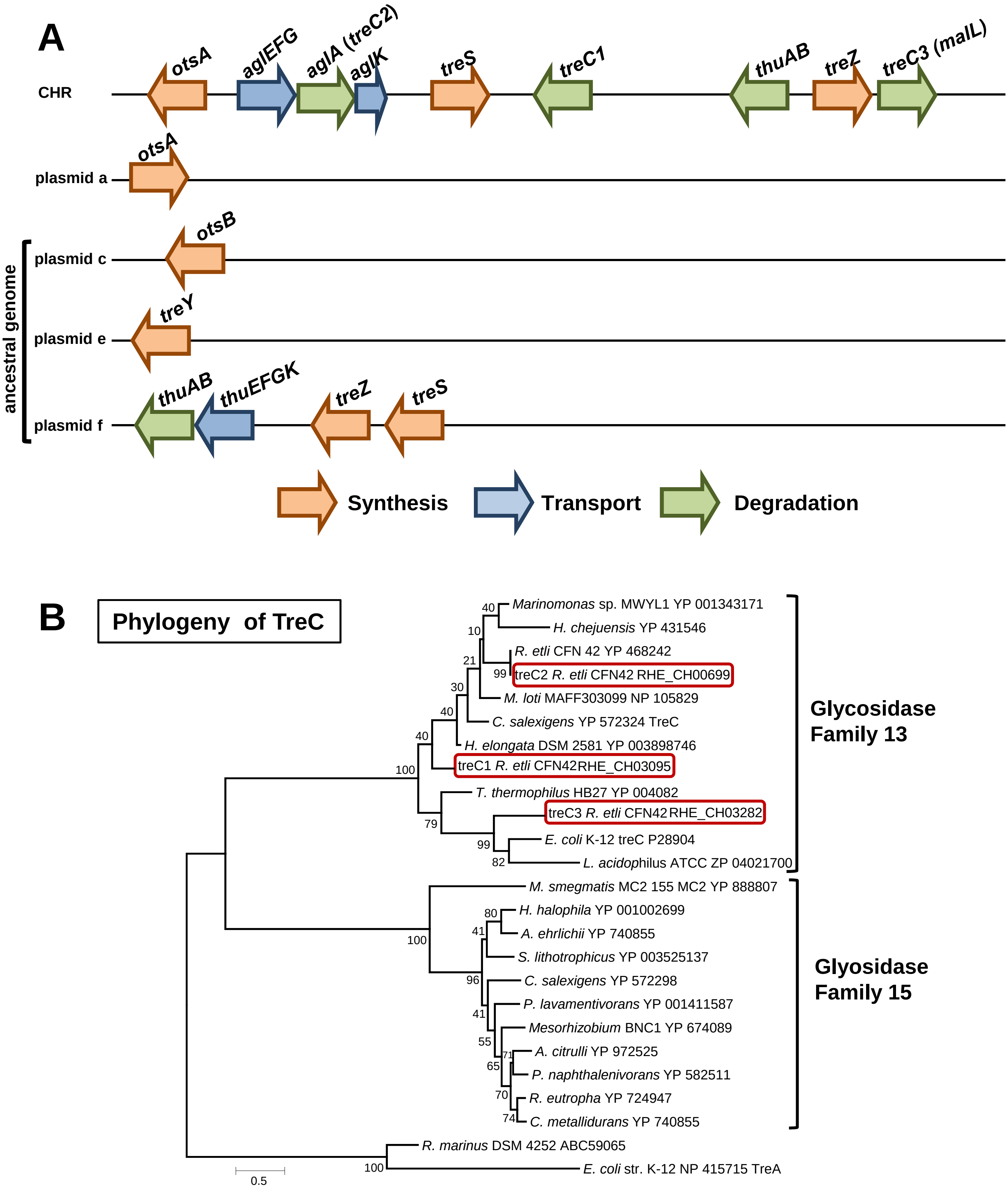

Figure S1. Genomic analysis of *R. etli* pathways involved in trehalose metabolism. (A) Genomic context of genes involved in trehalose metabolism. Position and clustering of genes included in Table S1 are indicated. (B) Neighbor-joining tree based on proteins belonging to families 13 and 15 of glycosydases, including the three TreC-like proteins from *R. etli*. The tree is drawn to scale, with branch lengths in the same units as those of the evolutionary distances used to infer the phylogenetic tree. The *E. coli* and *Rhrodothermus marinus* representatives were used as outgroup. The evolutionary distances were computed using the Poisson correction method and are in the units of the number of amino acid substitutions per site. The rate variation among sites was modeled with a gamma distribution (shape parameter = 1). All positions containing gaps and missing data were eliminated from the dataset (complete deletion option). Bootstrap probabilities (as percentage) were determined from 1000 resamplings.
