# Supplementary material for: Role of trehalose in heat and desiccation tolerance in the soil bacterium Rhizobium etli
Source: BMC Microbiol. 2012 Sep 17;12:207. doi: 10.1186/1471-2180-12-207 (PMC3518184; doi:10.1186/1471-2180-12-207)
Supplement: Additional file 3 — Figure S2. Growth of R. wild type (WT) and the otsAch mutant CMS310 with trehalose and glucose as the sole carbon source. Cells were grown in at 28°C in B- minimal medium with 20 mM trehalose or glucose and 0.0 or 0.2 M NaCl. [file 1471-2180-12-207-S3.pdf]

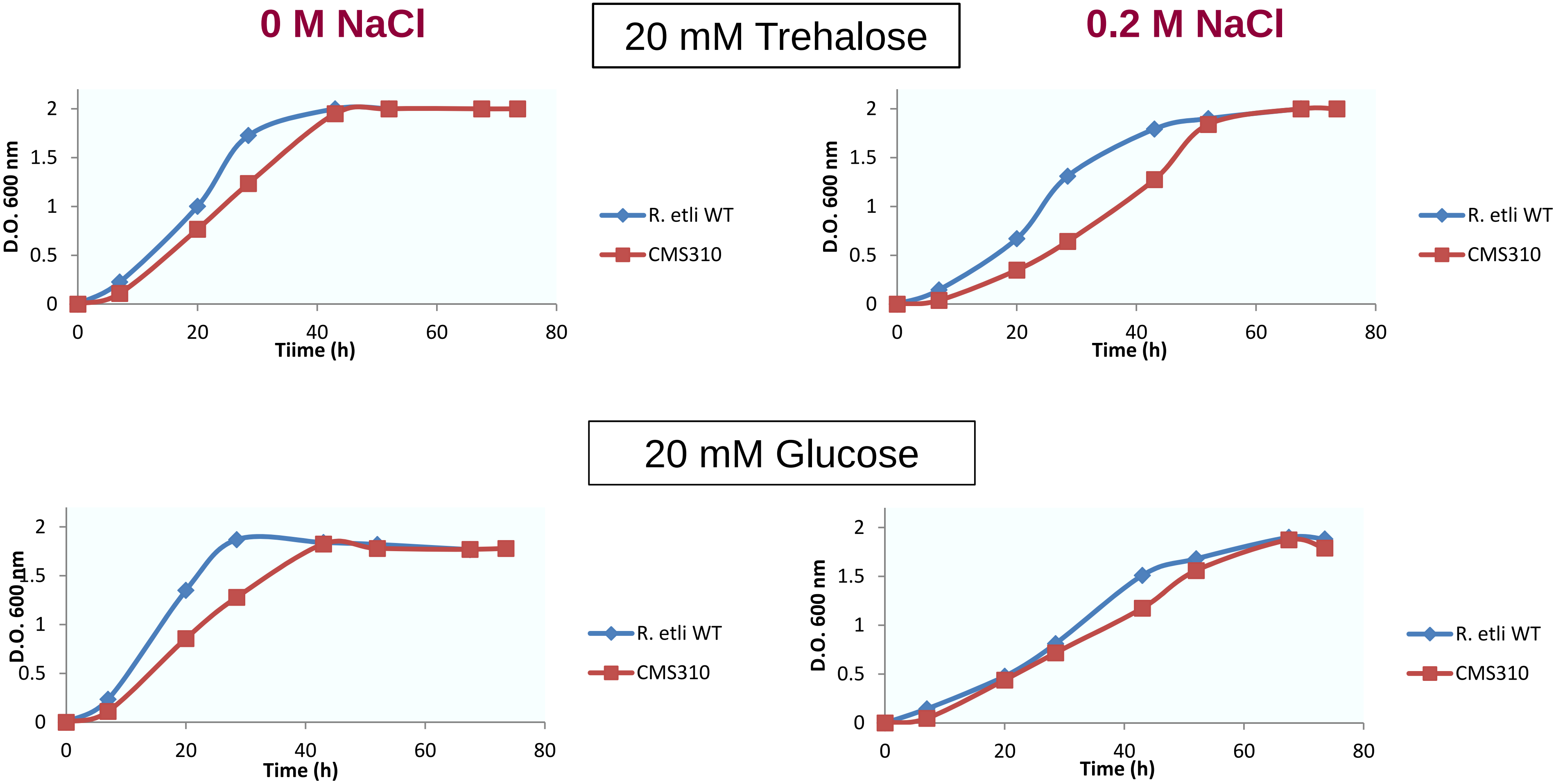

Figure S2. Growth of R. etli wild type (WT) and the *otsAch* mutant CMS310 with trehalose and glucose as the sole carbon source . Cells were grown in at 28 °C in B^- minimal medium with 20 mM trehalose or glucose and 0.0 or 0.2 M NaCl.
